# Convective Velocity Effects on a Thermistor in Water

**DOI:** 10.6028/jres.093.152

**Published:** 1988-10-01

**Authors:** Steve R. Domen

**Affiliations:** National Bureau of Standards Gaithersburg, MD 20899

**Keywords:** absorbed dose, calorimeter, convection, convective velocity, plume, point source heater, thermistor, water

## Abstract

Electrical powers from 5 to 150 μW were dissipated in a thermistor, causing it to rise to equilibrium temperatures above the stagnant surrounding water. Natural convection was then simulated by forced convection of water flowing up or down at known rates from 1.3 to 17 mm/min. The disturbances of the equilibrium temperatures were measured, and are presented as effects of equivalent absorbed dose and absorbed dose rates, positive and “negative.”

## 1. Introduction

Because water has absorption properties for ionizing radiation similar to biological tissue, it has been chosen as the standard reference material for radiation therapy [1]. Therefore, the absorbed dose^1^ water calorimeter was devised [2] and developed [3] for investigative studies concerning its use as a reliable standard for the calibration of instruments. One reason for the seemingly great delay in initiating such a calorimeter appears to have been concerns about the complexities of convection and its effect on the accuracy of measuring absorbed dose. Although convection at the point of measurement can be reduced or eliminated by the application of barriers [3] or by operating the calorimeter at 4 °C [4], it is of fundamental interest not only to better understand the water calorimeter, but also to understand convection in a system where there is essentially a free flow of water. The few observations reported (in an absorbed dose water calorimeter) are by the authors of reference [4] (who also operated their calorimeter at 30 °C) and by Barnett [5]. For a water calorimeter operated at ambient temperature or higher, it is valuable to know the best operational procedures to employ in order to reduce undesirable effects as much as possible. With the help of this knowledge and the use of convective barriers in the vicinity of the sensing thermistor, convective effects can be minimized to the point where they are likely to be insignificant.

A vast amount of theoretical and experimental work on convection has been reported in the field of fluid mechanics. The most successfully used instrument for measuring small velocities is the hotwire anemometer (1–3 mm long and 5–10 μm in diameter). However, the calibration of this instrument is difficult and special apparatus is required. The lowest velocity at which it has been calibrated was about 8 mm/min [6]. A later work is also described [7], and several difficulties of its use in water are pointed out [8]. Depending on irradiation conditions and position of measurement in a barrier-free water calorimeter, the convective velocity could vary from zero to about 10 mm/min. Even if a small velocity could be measured accurately, there would still remain the important problem of determining the effects of convection on the accuracy of measurement of absorbed dose with a temperature-calibrated thermistor. The equilibrium temperature rise could be significantly disrupted by the motion of the water.

To circumvent the above difficulties, a thermistor was used both as a temperature-measuring device and as a velocity-measuring device. Its velocity sensitivity is significantly greater than that of the hot-wire anemometer.

In the present investigation, natural convection was simulated by forced convection of water flowing around a thermistor over a wide range of known water velocities and thermistor powers. The changes in thermistor temperature were measured and are presented as the effect of an equivalent absorbed dose or absorbed dose rate, quantities of direct interest to those in the field of radiation dosimetry. A conversion factor can be used to rescale the results in terms of temperature.

## 2. Sources of Convective Effects

Dissipation of electrical power in the thermistor will cause it to rise to an equilibrium temperature above that of the surrounding stagnant water, which might be either uniform or nonuniform in temperature. Irradiation of the water will produce temperature gradients which are the driving force of convection, and which may set it into motion.

Measurement errors due to convection as a result of irradiation may then arise in two ways: (1) if the background temperature of the water is uniform, the motion of the water may disturb the equilibrium temperature rise of the thermistor, and (2) if the background temperature of the water is nonuniform, an additional disturbance may result from a temperature difference between the water entering and leaving the region of the thermistor. The study of effect (1) is the main purpose of this investigation. Applications will be described elsewhere [9].

Nonuniform temperature distribution in the water may have two causes: (a) a significant variation of absorbed dose rate in the vicinity of the thermistor from previous runs, and (b) conduction of heat between the calorimeter and surroundings. Cause (b) can be reduced by placing an enclosure around the calorimeter to circulate the air and regulate it within 0.1 °C. Such a simple enclosure was used and has been previously recommended [3]. Any remaining temperature gradients, caused by either (a) or (b), can be rapidly and effectively erased by simply agitating and circulating the water between runs. A significant observed drift is a good indication that this needs to be done.

## 3. Setup

**Figure 1:**
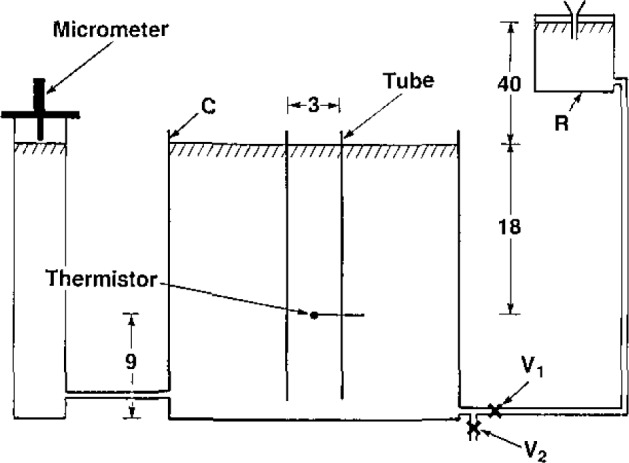
Experimental setup for simulating water convection to study the change in thermistor equilibrium temperature and the resulting effect on a measurement of absorbed dose or absorbed dose rate. The drawing is not to scale. The dimensions are in centimeters.

Figure 1 shows a sketch of the experimental setup. A calibrated thermistor (which formed one arm of a Wheatstone bridge) was positioned on the vertical axis of a two-piece plastic tube, 3 cm inside diameter. The tube was supported in a calorimeter container C filled with once-distilled water. Plastic tubes connected C to an elevated water reservoir R and a column of water where micrometer measurements were made to its surface.

C was surrounded by thick insulation consisting of expanded polystyrene (for further details see figs. 3 and 5 in [3]). The desired uniform temperature in C was achieved with immersion heaters and circulating the water with air, which bubbled upward along the four vertical edges of C. Thermistor probes in C, R, and the room sensed temperatures, which were displayed with a resolution of 0.01 °C.

The large cross-sectional area of R (1,400 cm^2^), helped to maintain a sufficiently constant flow of water. The instantaneous initial and final surface velocities *S_v_* in C differed by 1 to 5%, as a result of changes in water level heights (about 3 to 13 mm in C).

Measurements were made of the average surface velocities ${\bar{S}}_{v}$, rise or fall. This was then used to determine the average axial water velocity ${\bar{A}}_{v}$ along the tube axis. (This is discussed below in section 6, ${\bar{S}}_{v}\neq {\bar{A}}_{v}$). ${\bar{S}}_{v}$ ranged from 1.3 to 10 mm/min. ${\bar{S}}_{v}$ (rise) was determined from the initial and final micrometer measurements and a measured time interval, from the time when the ball valve V_1_ was suddenly opened to when it was closed. ${\bar{S}}_{v}$ (fall) was determined in a similar manner but by operating ball valve V_2_; and, instead of the micrometer measurements, it was determined from the measured mass of water withdrawn and the cross-sectional areas of C (927 cm^2^) and the water column (16 cm^2^). The water lines contained other valves (not shown), which were pre-adjusted to give desired rates of water flow when V_1_ or V_2_ was opened.

## 4. Preparation

Initially the water was circulated with air and the immersion heaters were turned on until the water was heated to room temperature (regulated within about ±0.2 °C). This procedure minimized subsequent internal temperature drifts. The temperature of the stagnant water within the tube lagged behind that of the surrounding agitated water. To speed approach to uniformity of temperature of the entire water volume and of the two-piece tube, the upper part of the tube was detached (a few centimeters above the thermistor plane) and placed in a comer of C where water flowed vigorously within and without that part of the tube. At the same time, the motion of the water reduced the time to achieve uniformity of temperature in the lower part of the tube (still in position). About 30 minutes later the two parts were rejoined. Several minutes later the air supply was turned off and the water became stagnant. During the above procedure, the agitated water in R had been adjusted in temperature until it was observed to be the same as that in C.

## 5. Procedure

To assure repeatability of water velocities, the heights of the water levels in C and R were restored prior to a run. For example, to simulate upward convection, V_1_ was opened, which caused water levels in C and R to rise and fall, respectively. After the run, the amount of water that had entered C was withdrawn through V_2_ and replaced in R. To simulate downward convection, V_2_ was opened, lowering the water level in C. The water withdrawn was then poured into R, and V_1_ was opened until the water levels were restored.

A procedure was also followed to assure temperature stability as much as possible. If successive runs were made, following the above procedure alone, increased temperature instabilities would result because of small temperature differences of the water flowing into C. This would result in (1) temperature drifts caused by heat conducted toward or away from the tube axis, and (2) a temperature gradient along the tube axis, which is of more concern. Therefore, when the levels were restored after each run, the water in C was agitated for several minutes. This assured an essentially uniform temperature distribution along the outside of the tube and an eventually more uniform temperature along the tube axis. The motion of the water, however, did cause a significant disturbance in the thermistor equilibrium temperature, but after agitation equilibrium was restored within 10 minutes.

It is, however, still desirable to reduce the relatively uniform rate of heat conduction to or away from the entire tube axis. The direction of the observed drift (cooling or heating) indicated that R needed a small amount of warm or cool water, followed by agitation; or an eyedropper of hot or ice water was directly injected into C and agitated. The result was a sluggish and delayed reaction, but nevertheless this procedure did significantly help to provide thermal stability. The remaining small drift signals were quickly reduced by making an adjustment with a resistance-capacitance circuit across the Wheatstone bridge [10]. As with other calorimeters, this circuit proved effective in producing an essentially zero initial drift signal.

## 6. Axial Water Velocity

In figure 1, the water surfaces (inside and outside the tube) rise or fall at the same rate, *S_v_*. Although *S_v_* will slowly change (because of the changing heights with respect to V_1_ and V_2_), we consider the ideal situation where *S_v_* is constant.

During the slow laminar flow of a fluid in a tube, the velocity at the wall remains zero. As time increases from *t*=0, the velocity increases toward and along the tube axis, where it is greatest. At positions along the axis (sufficiently far from “end effects” of the water surface and the bottom end of the tube), the instantaneous axial velocity *A_v_* will increase from *S_v_* to 2*S_v_*. The vertical velocities within the tube build up to a parabolic distribution, as a function of radius, A detailed theoretical analysis is given by Szymanski [11]. A figure and a detailed table are presented in reference [11] which give the buildup velocity profiles as a Function of time and radius. The figure is also presented in a more recent reference [12], p. 129. Reference [13], among many others, gives additional detailed information on fluid flow. Laminar flow occurs when the Reynolds number

$$Re=\frac{DV}{v}<2000.$$ (1)

In equation (1) *D*=inside diameter, *V*=average velocity within the tube (*V=S_v_*), *S_v_*=surface velocity, and *v*=kinematic viscosity. For the present experiment, *D*=3 cm, *S_v_*=1 cm/min (the maximum velocity used), and *v*=0.59 cm^2^/min (at 21 °C, the operating temperature of the calorimeter). Substituting these values in equation (1) gives *Re* ≃ 5, well within the range for laminar flow.

In general, the velocity on the axis for any size tube varies approximately linearly with time from the initial value *S_v_* up to 1.5*S_v_*. For the 3-cm- diameter tube used in this experiment, the theoretical times required for the buildup to reach 50 and 95% of the maximum are 0.53 and 2.1 min, respectively. Therefore, if at *t*=0 the surface velocity is *S_v_* (and remains constant), then at those respective times the velocities on the central axis are 1.50*S_v_* and 1.95*S_v_*. In reality (as mentioned previously) ${\bar{S}}_{v}$ is measured, In the data analysis, ${\bar{S}}_{v}$ is treated as an assumed constant *S_v_*. Therefore, only *S_v_* will be mentioned in the remainder of this paper.

The equilibrium temperature of the thermistor was, therefore, disturbed by a steady increase in axial water velocity. One phase of the measurements required measuring the average rate of temperature change in the thermistor (which can be considered as the effect of an equivalent absorbed dose rate) during a measured time interval, This was related to ${\bar{A}}_{v}$ during that interval. ${\bar{A}}_{v}$ was computed by use of *S_v_* and an empirical fit to the theoretical data mentioned in reference [11], integrated over the time interval.

## 7. The Thermistor

The thermistor indicated in figure 1 is a commercially available, electrically insulated thermistor (uninsulated thermistors were extremely noisy). The nominal size of the bead was 0.25 mm. It bad two platinum leads (18 μm in diameter, 1 mm long) soldered to paired nickel alloy leads (76 μm in diameter). The bead and the 18-μm diameter wires had a thin coating of resin, of variable thickness <25 μm. An improvement was made by applying a thin coat of silicone rubber which significantly increased the electrical resistance between those components and the surrounding water. The resulting outside diameter of the silicone coating around the bead was nearly 0.35 mm. The diameter of the commercial insulating sleeve around the paired 76- μm wires was 0.23 mm.

**Figure 2:**
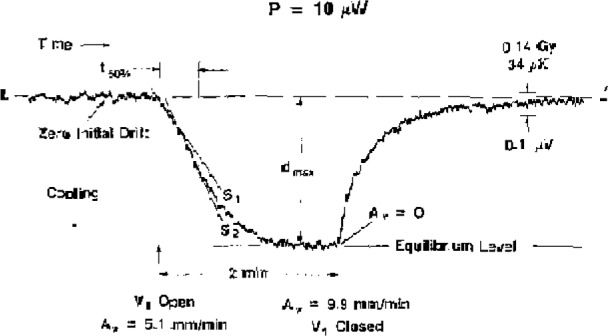
Typical response of a thermistor as a result of upward convection.

The performance of the thermistor was good throughout the experiment. The recordings (shown in figure 2 and subsequent figures) indicate good signal-to-noise ratios. After each day of measurement, the water in container C was drained and the support tube and its thermistor were removed and dried in the air-conditioned room.

At a calorimeter operating temperature of 21 °C, the thermistor resistance was 2.6 kΩ and its measured resistance sensitivity was 3.7%/°C. From the measurements of its resistance as a function of temperature and power, the equilibrium temperature rise (Δ*T*) of the thermistor above that of the surrounding stagnant water was:

ΔT=1.41PmK, (2)

where *P*=power in microwatts.

The value of Δ*T* could be significantly different for a different bead size and details of insulation. Measured results of convective effects are expected to vary directly with Δ*T*. Therefore, an experimenter should have a similar knowledge of a thermistor being used, in order to apply the results of this investigation. In general, it is good practice for experimenters to give an expression similar to eq (2) along with the thermistor powers which were used. Otherwise, the meaning of some reported results may be unconvincing to the readers.

The temperature calibration of the thermistor was also determined from the measurements of its resistance as a function of its temperature and power. This permitted calorimeter responses to be converted to temperature changes, described previously in detail [3].

## 8. Results

### 8.1 Upward Convection

Figure 2 shows a typical general response as a result of forced upward convection. In all cases, it was possible to preset the power to within an uncertainty of 0.1% and to produce an essentially zero initial drift. Valve V_1_ was then suddenly opened, causing *S_v_=A_v_*=5.1 mm/min. Convective cooling of the thermistor was instantaneous, because of its increased heat-transfer coefficient ([12], p. 409). Its cooling rate depended on *A_v_* and power *P*, 10 μW for the run shown in figure 2. After 2 minutes, for this case, the thermistor response appears to be near equilibrium. At this time, the theoretical velocity buildup reached 94% of the maximum [11], giving a value of *A_v_*=1.94*S*, =9.9 mm/min. Then V_1_ was closed (rapidly causing *A_v_* to drop to 0), which caused the thermistor to rise in temperature to its initial equilibrium value represented by the base line LL′. This is an indication that the background temperature of the water which left the vicinity of the thermistor was equal to that which entered that region. When this is not the case, the final trace will undershoot or overshoot LL′. Large effects like this were avoided by frequent agitation of the water and the preparation procedure (described in sections 1 and 4) to assure more uniformity in background temperature. Corrections were made for any of those small remaining effects caused by nonuniformity.

The scale on the right in figure 2 indicates the measurement sensitivity. The indicated absorbed dose or temperature change would have caused a 0.1-μV potential change in the output of the Wheatstone bridge, which was powered by a 1.35-volt mercury cell. The noise shown is approximately the greatest encountered for all values of power.

The thermistor response during water flow showed a wide range of characteristics, which varied with power and water velocity. Figure 2 shows an initial slope S_1_ followed by a steeper slope S_2_. Particularly at large signals caused by high powers (when the gain of the amplifier was reduced, resulting in little or no observable noise fluctuations), the transitional point from S_1_ to S_2_ in many cases appeared to be clear and abrupt; under some conditions there was more than one point of inflexion. Also, in many cases S_1_>S_2_. The time to reach apparent equilibrium varied significantly.

The somewhat complicated response indicated by the recordings required some compromises in presenting the measured results. The recordings show two main characteristics: (1) a rate of change of response, and (2) a maximum deflection *d*_max_, represented by the indicated equilibrium level. At *d*_max_ the value of *A_v_* assigned was *2S_v_*. The quotient of *d*_max_/2 divided by the corresponding time interval is a measure of the rate of response up to 50% of *d*_max_. If *d*_max_ is expressed in terms of equivalent dose, then that quotient has the dimensions of a dose rate. While strictly speaking it is not an “average,” it is in fact close to a true average, and it will be convenient here to refer to it as the average dose rate during the time interval *t*_50%_. That time interval appeared to be a reasonable choice, because beyond that point the signal began to decrease rapidly. The equivalent “negative” average absorbed dose rates *Ḋ*^−^ are plotted as a function of ${\bar{A}}_{v}$ during the time interval *t*_50%_.

A 1 mK rise in temperature is produced by an absorbed dose of 4.18 Gy, neglecting a heat defect. This conversion factor can be used to rescale the plotted results in terms of temperature.

**Figure 3:**
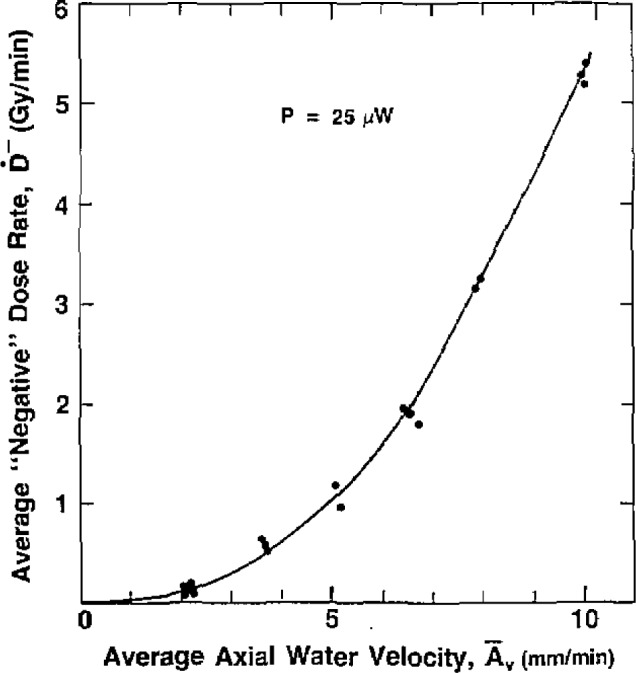
Plot of a daily set of measurements. The average “negative” absorbed dose rate *Ḋ*^−^, from 0 to 50% of equilibrium, is plotted as a function of the average axial water velocity ${\bar{A}}_{v}$, during the interval, *t*_50%_.

**Figure 4:**
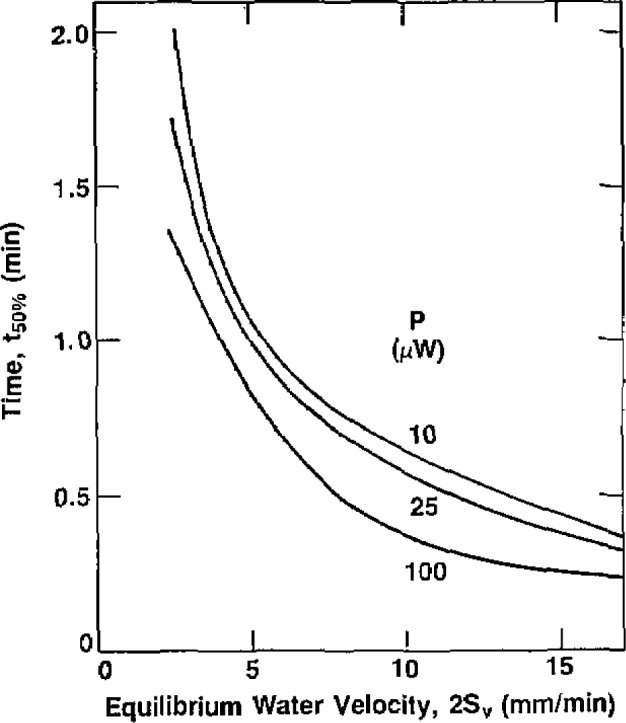
Plots of the time *t*_50%_ for the thermistor to reach 50% of its drop to equilibrium as a function of the equilibrium water velocity 2*S_v_* along the tube axis.

Figure 3 shows a plot of individual points determined during a daily set of measurements, for *P*=25 *μ*W. The average *Ḋ*^−^, from 0 to 50% of equilibrium, is plotted as a function of the average axial water velocity. (The plots in the figures that follow do not show the individual points of measurements, for the sake of clarity.) Figure 4 shows plots of the approximate times for the thermistor to reach 50% of its temperature drop to equilibrium as a function of the equilibrium axial water velocity 2*S_v_*.

**Figure 5:**
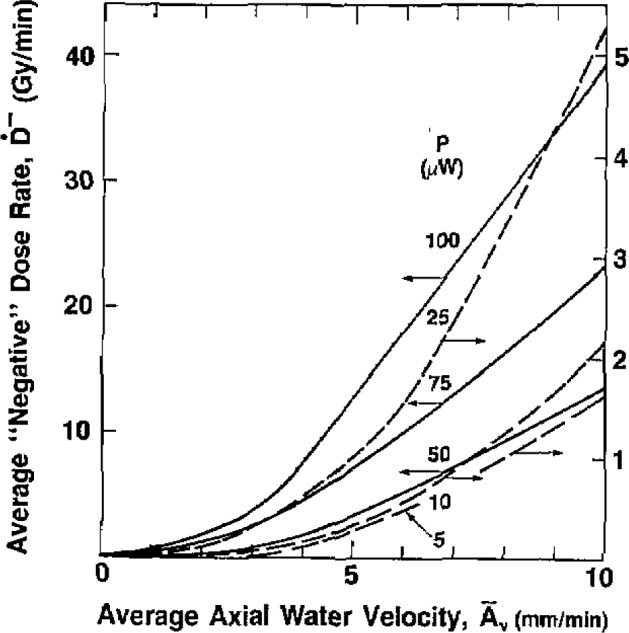
Summary of the measurements of the average “negative” absorbed dose rate *Ḋ*^−^ as a function of the average axial water velocity $\bar{A}$ during the time interval *t*_50%_.

**Figure 6:**
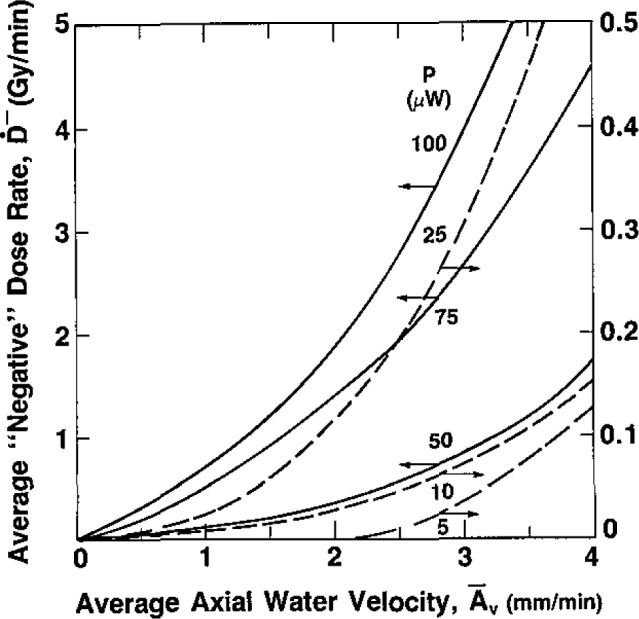
Expanded scale, from ${\bar{A}}_{v}=0$ to 4 mm/min, of the plot shown in figure 5.

**Figure 7:**
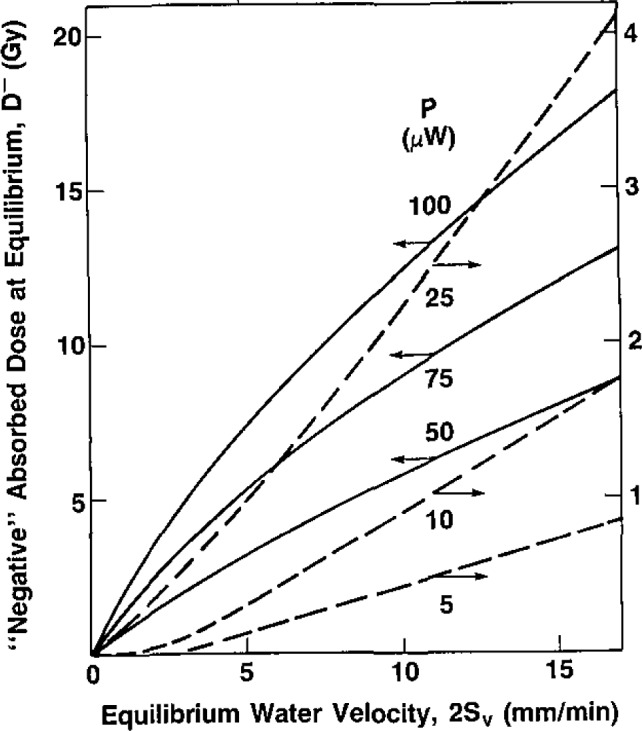
Summary of measurements of the equivalent “negative” absorbed dose *D*^−^ at equilibrium as a function of the equilibrium water velocity 2*S_v_* along the tube axis.

Summaries of the measurements (for *P* = 5 to 100 *μ*W) are shown in figures 5, 6, and 7. An expanded scale of figure 5 (from ${\bar{A}}_{v}=0$ to 4 mm/min) is shown in figure 6. At low values of *P* and ${\bar{A}}_{v}$, *Ḋ*^−^ is very small. Therefore, the curves in those regions should serve only as a guide, because some subjectivity was used in extending the curves to zero ${\bar{A}}_{v}$ and *Ḋ*^−^. Figure 7 shows plots of the equivalent “negative” absorbed dose *D*^−^ at equilibrium as a function of the equilibrium water velocity 2*S_v_* along the tube axis.

The smallest values of surface velocity and power were 1.3 mm/min and 5 *μ*W, respectively. Six 2-minute runs were made in this condition. There appeared to be no detectable response to about 1.5 minutes. At the end of the run (*A_v_* = 2.5 mm/min), *D*^−^ was perhaps about 0.04 Gy. This was barely detectable, being just above the noise level. Figures 5 and 6 can be used in practice to estimate roughly the convective velocity produced by irradiation, by use of the initial and final drift rates of a recorded calorimeter run [9].

### 8.2 Downward Convection

The investigation of downward convection started with initial values of *S_v_* = 3.5 mm/min and *P*=30 μW. The convective cooling of the thermistor had the same general shape as that shown for upward convection in figure 2. The same was noted when *P*=40 μW. Most of the time this appeared to be the case for *P*=50 μW, but occasionally (after V_2_ was opened) there appeared to be a short and barely detectable heating response before convective cooling began. At *P*=60 μW the convective heating was clearly visible on nearly all occasions. It always appeared at higher values of power, and the response and duration increased with power. The power was varied up to 150 μW.

**Figure 8:**
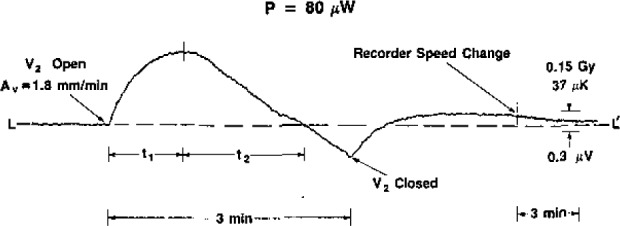
Thermistor response to downward convection, *P*=80 μW and *A_v_*=1.8 mm/min at *t*=0.

**Figure 9:**
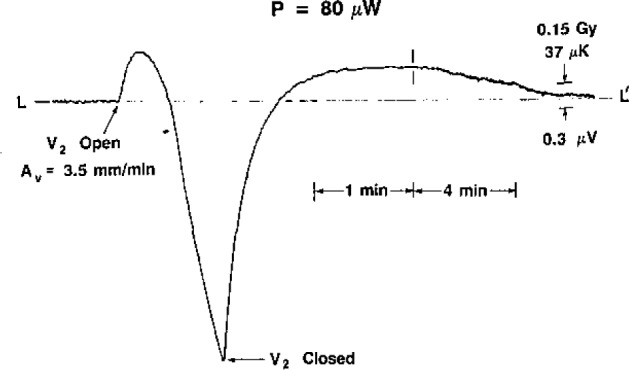
Thermistor response to downward convection, *P* = 80 μW and *A_v_* = 3.5 mm/min at *t*=0.

**Figure 10:**
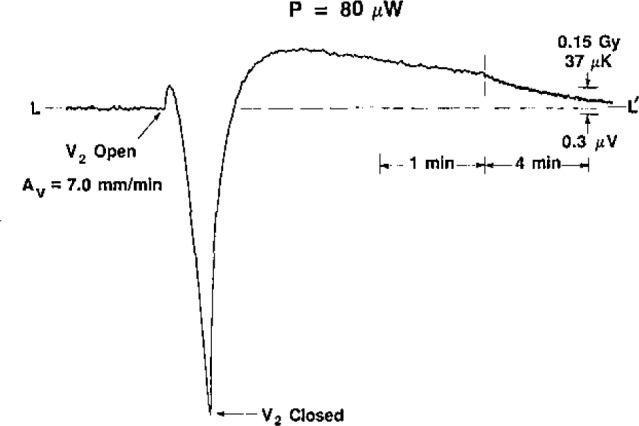
Thermistor response to downward convection, *P*=80 μW and *A_v_*=7.0 mm/min at *t*=0.

**Figure 11:**
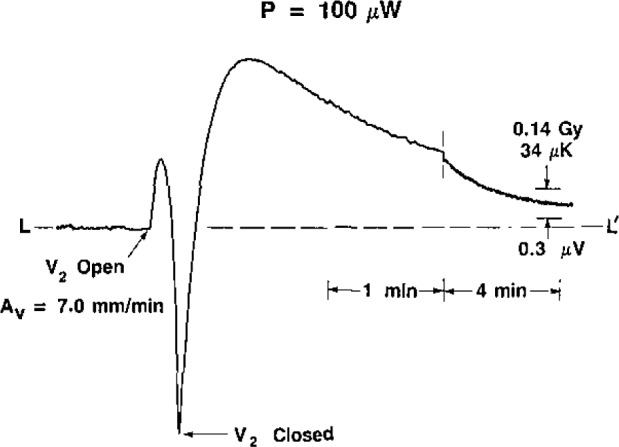
Thermistor response to downward convection, *P*= 100 μW and *A_v_*=7.0 mm/min at *t*=0.

Figures 8, 9, and 10 show the development of the recordings when the initial velocity was varied, *P*=80 μW. Figure 10 may be compared with figure 11, where *P* = 100 μW.

**Figure 12:**
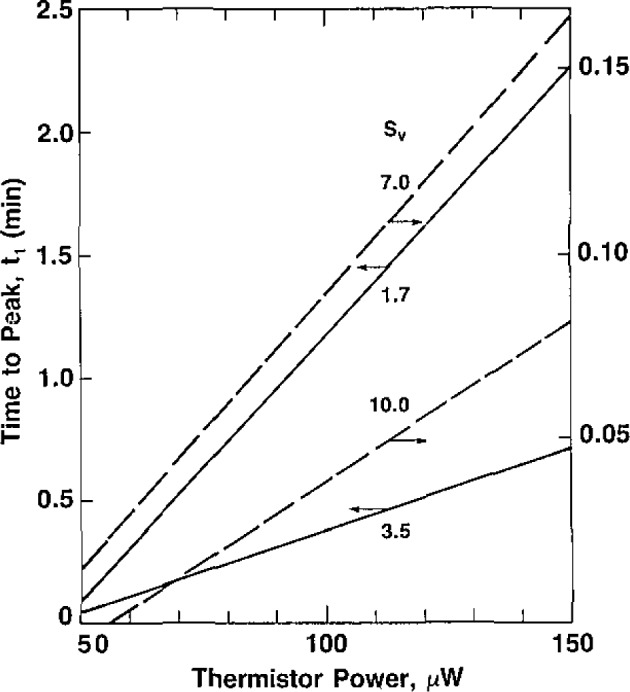
Summary of the measurements of the time *t*_1_ to the peak of the temperature rise as a function of thermistor power. The surface velocity *S_v_* is in mm/min.

**Figure 13:**
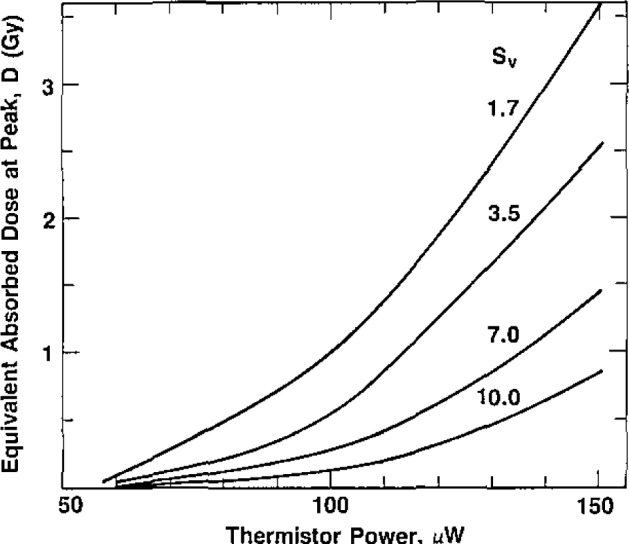
Summary of measurements of the equivalent absorbed dose *D* at the peak, at the end of time *t*_1_, as a function of thermistor power. The surface velocity *S_v_* is in mm/min.

Figures 12 and 13 show, respectively, summary measurements of the time interval *t*_1_ and the equivalent absorbed dose *D* at the peak of response at the end of *t*_1_, (see fig. 8) as a function of power. Figures 12 and 13 can be used for estimating the average equivalent absorbed dose rates *Ḋ* during *t*_1_.

**Figure 14:**
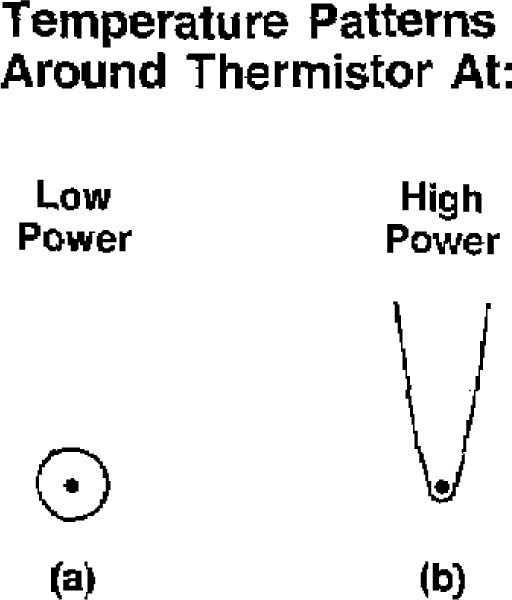
Illustrations of the equilibrium temperature patterns around a thermistor.

A reason for the convective heating is postulated by use of the illustrations in figure 14. At low thermistor powers, figure 14(a), the temperature pattern around the thermistor is essentially symmetric, implying no or little convection. Movement of that pattern, either up or down, would increase the net temperature gradient in the immediate vicinity of the thermistor to cause it to cool. As the power is increased, *localized* convection will eventually occur, giving rise to a plume, figure 14(b). Forced upward convection would further increase the net temperature gradient, since the bottom of the plume would be forced nearer to the thermistor, and it would be expected that convective cooling of the thermistor would be observed. Downward convection will cause the plume to move down relative to the thermistor. This will initially result in a net decrease in temperature gradient, since the bottom end of the plume will be at a greater distance from the thermistor. This will decrease its heat transfer coefficient, causing the thermistor at least initially to rise higher in temperature, The magnitude of this effect will depend on the axial velocity, At a low velocity a longer time will be required for the plume to pass through the thermistor, causing a relatively long duration of convective heating.

By the time the value V_2_ was closed (figs. 8 to 11), the plume, figure 14(b), had descended downward, which caused heating of the water over an extended distance below the thermistor. This volume of water then ascended upward to cause a temperature overshoot before temperature equilibrium LL′ was attained.

**Figure 15:**
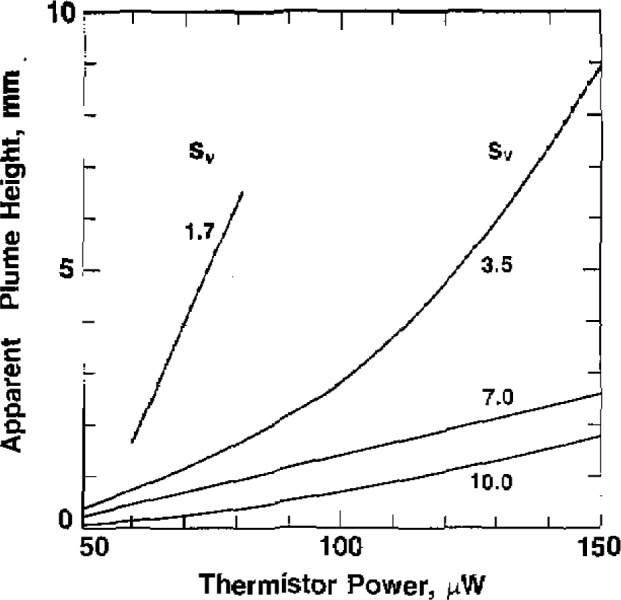
Summary of measurements of the “apparent” plume height as a Function of thermistor power. The surface velocity *S_v_* is in mm/min.

Figure 15 shows plots of the apparent plume height as a function of power. This is the product of *t*_1_+*t*_2_ and ${\bar{A}}_{v}$ during that interval. For a given value of power, the results show a wide variation in the apparent plume heights. When *S_v_*=1.7 mm/min, the runs from 100 to 150 μW showed that the signal was well above the base line LL′, when V_2_ was closed. The results of those runs could not be shown, because they do not have the required time interval *t*_1_+*t*_2_. When *S_v_*=3.5 mm/min, a large change in effect was seen. The change is still significant when *S_v_*=7.0 mm/min. A still significant but smaller change was noted when *S_v_*=10.0 mm/min.

It can be seen that the curves in figure 15 do not converge rapidly. Therefore, no definite conclusions can be drawn from this information, which is presented merely to show this type of behavior.

The above described test for convection around a thermistor as a function of power is much more sensitive than that previously described [3], where it was concluded that no convection of that type was observed.

#### 8.2.1. Plume Theory

The characteristics of a plume from a point heat source have been derived theoretically [14]. This is also discussed in [15], pp. 353–354 and [8], pp. 110–115. The basic theoretical equations apply to the condition where the rate of heat transfer from the source is sufficiently high to create a laminar convective plume. The present experiment indicates that this condition is satisfied when the power is at least 50 μW.

The theoretical calculations predict the following for a point source in water and *P*=100 *μ*W The velocity along the vertical axis is 7.2 mm/min, which remains constant with *x*, the distance above the source. The temperature rise at *x*=1 mm is 1.5 mK. Generally, the velocity and temperature rise vary as *P*^1/2^ and *Px*^−1^, respectively. The plume radius varies as *x*^1/2^ *P*^−1/4^. The rate of water flow through a horizontal plane at *x*=1 mm is 1.1 g/min; the mass flow rate varies as *x*, independent of *P*. This surprising result is due to the fact that mass flow is proportional to the product of velocity and the square of the radius, which is seen to be proportional to *x* [14].

## 9. Conclusion

The results of this investigation clearly reveal a potential source of inaccuracy in measurement of absorbed dose using large thermistor powers, in a water calorimeter in which uninhibited convection is allowed in the vicinity of the thermistor. Figures 5 and 6 can be used to estimate attenuation of this error by inhibiting the convective velocity with barriers and reducing the thermistor power, which should perhaps be as low as 2 μW. The output sensitivity of a Wheatstone bridge varies as the square root of the power, so the sensitivity does not decrease as rapidly as the power. Good signal-to- noise ratios can be attained at low power levels, particularly with the high dose rates available with therapy accelerators. To test the initial choice of thermistor power and convective barrier geometry, preliminary irradiation measurements should be made at several different power levels. No variation in measurements should be observed.

## 10. Summary

A calibrated thermistor was immersed in water. Electrical power dissipated in the thermistor caused it to rise to an equilibrium temperature above its initial temperature. The power was varied from 5 to 150 *μ*W, and the equilibrium temperatures were disturbed by forced downward and upward simulated convection with water velocities from 1.3 to 17 mm/min. The velocities were related to the rate of temperature change, and also to the total temperature change from the original equilibrium value to a new equilibrium value. The rate of temperature change and temperature change were expressed as equivalent absorbed dose rate and absorbed dose, either positive or “negative.”

## Footnotes

1 Absorbed dose is the energy per unit mass imparted to matter by ionizing radiation. The SI unit of absorbed dose is the gray (symbol Gy). 1 Gy = 1 J/kg.

## References

[1] (1969). ICRU Rep No 14, Appendix B.

[2] Domen SR (1980). Absorbed Dose Water Calorimeter. Med Phys.

[3] Domen SR (1982). An Absorbed Dose Water Calorimeter: Theory, Design, and Performance. J Res Natl Bur Stand (US).

[4] Schulz RJ, Weinhous MS (1985). Convection Currents in a Water Calorimeter. Phys Med Biol.

[5] Barnett RB (1986). Water Calorimetry for Radiation Dosimetry. PhD Thesis.

[6] Dring RP, Gebhart B (1969). Hot-wire Anemometer Calibration for Measurements at Very Low Velocity. J Heat Transfer.

[7] Hollasch K, Gebhart B (1972). Calibration of Constant- Temperature Hot-Wire Anemometers at Low Velocities in Water with Variable Fluid Temperature. J Heat Transfer.

[8] Jaluria Y (1980). Natural Convection Heat Mass Transfer.

[9] Domen SR Further Comments on Convection Currents in a Water Calorimeter. Phys Med Biol.

[10] Domen SR (1983). A Temperature-Drift Balancer for Calorimetry. Int J Radiat Isot.

[11] Szymanski P (1932). Quelques Solutions Exactes des Équations de l’Hydrodynamique du Fluide Visqueux dans le Cas d’un Tube Cylindrique. J Math Pures Appl, Series 9.

[12] Bird RB, Stewart WE, Lightfoot EN (1960). Transport Phenomena.

[13] Hall NA (1951). Fluid Friction, Chapter 2 in Thermodynamics of Fluid Flow.

[14] Fujii T (1963). Theory of the Steady Laminar Natural Convection Above a Horizontal Line Heat Source and a Point Source. Int J Heat Mass Transfer.

[15] Gebhart B (1971). Heat Transfer.

